# An Optimal Receiver Deployment Method for Achieving Robust Localization Performance in Practical Environments

**DOI:** 10.3390/s26175486

**Published:** 2026-08-29

**Authors:** Bosung Park, Jongho Keun, Hosung Choo

**Affiliations:** Department of Electronic and Electrical Engineering, Hongik University, Seoul 04066, Republic of Korea; midalli324@mail.hongik.ac.kr (B.P.); antkeun@mail.hongik.ac.kr (J.K.)

**Keywords:** AoA-based localization, receiver deployment optimization, PDoP, propagation environment, ray-tracing method, robust localization

## Abstract

In this paper, we propose a receiver deployment optimization method that ensures robust localization performance under Direction of Arrival (DoA) estimation errors arising in practical environments. The proposed method accounts for DoA estimation errors and optimizes receiver deployments by incorporating Position Dilution of Precision (PDoP) to mitigate their amplification into localization errors. To accurately characterize DoA estimation errors under realistic propagation and installation constraints, a ray-tracing-based Wireless InSite simulator is employed to model environments including actual terrain and buildings. Based on this model, a cost function incorporating both the DoA estimation errors at each receiver and the PDoP is defined, and a Genetic Algorithm (GA) is applied to minimize this cost function and determine the optimal receiver deployment. The optimal deployment achieves low localization RMSE and maintains high robustness against environmental and system uncertainties, particularly when the standard deviation of the DoA estimation error exceeds approximately 8°. These results demonstrate that the proposed deployment method provides reliable localization performance in realistic propagation environments and remains robust against increasing DoA estimation errors.

## 1. Introduction

Over the past few decades, satellite communication systems have become essential infrastructure in both military and civilian applications, including broadcasting, remote sensing, and navigation [1,2,3]. As the importance of these systems has increased, they have also become prime targets for intentional interference, leading to a growing number of jamming attempts [4,5,6]. In particular, such intentional jamming can be carried out by highly mobile platforms, such as Unmanned Aerial Vehicles (UAVs), which can emit jamming signals from various locations [7,8,9]. In response to jammers operating in such unpredictable locations, localization systems should be deployed near communication facilities to accurately estimate the location of the jammer based on received jamming signals. Various localization techniques, including Time of Arrival (TOA), Received Signal Strength (RSS), Frequency Difference of Arrival (FDOA), and Angle of Arrival (AoA), have been investigated for localization. Among them, TOA-, RSS-, and FDOA-based localization methods require additional signal information or specific conditions, such as accurate timing synchronization, reliable received power information, or sufficient relative motion, respectively [10,11,12]. In contrast, AoA-based localization is widely adopted in localization applications because it does not require such information or conditions. In AoA-based localization, multiple array receivers estimate the Direction of Arrival (DoA) of incoming jamming signals and determine the jammer position through triangulation [13,14]. However, in practical environments, DoA estimation errors inevitably arise due to various factors, including environmental effects such as multipath propagation caused by reflection and diffraction, as well as receiver-related factors such as receiver hardware characteristics and operational constraints [15,16]. Moreover, these inevitable DoA estimation errors can be significantly amplified into localization errors depending on the geometric deployment of receivers, which is commonly characterized by the Position Dilution of Precision (PDoP) [17,18,19]. Accordingly, achieving robust localization performance under such unavoidable DoA estimation errors is critical for the reliable operation of localization systems in practical environments. To address these challenges, numerous studies have investigated DoA estimation errors under noisy environments and system losses. Conventional approaches have analyzed the performance of DoA estimation algorithms, such as Capon, MUSIC, and ESPRIT, and proposed methods to mitigate their sensitivity to noise and model errors [20,21,22,23]. In addition, recent studies have addressed the compensation of practical receiver- and processing-related artifacts in AoA estimation through phase-error correction and compensation techniques, while low-complexity approaches based on 1-bit conversion have been investigated for real-time AoA estimation. These approaches can mitigate receiver-related and processing-induced errors arising from hardware imperfections and practical implementation constraints [24,25,26,27]. However, these studies mainly focus on receiver and processing-related error factors and their correction or compensation, while realistic environmental effects such as multipath propagation are not sufficiently considered. More recently, machine learning-based approaches have been proposed to improve DoA estimation performance by learning complex receiving signal characteristics in realistic environments [28,29]. Nevertheless, these studies mainly focus on reducing DoA estimation errors and do not consider how such errors can be amplified into localization errors depending on the geometric deployment of the receivers. Although several receiver deployment approaches have been proposed based on geometric metrics, direct localization-error minimization, or environment-aware optimization, they do not explicitly consider the geometric amplification of practical DoA estimation errors in receiver deployment [30,31,32]. Table 1 summarizes the main differences between these representative approaches and the proposed method. Therefore, achieving robust localization performance requires an optimal receiver deployment that sufficiently accounts for DoA estimation errors arising from practical environments while minimizing their amplification into localization errors.

In this paper, we propose a receiver deployment optimization method that ensures robust localization performance under DoA estimation errors arising in practical environments. The proposed method accounts for DoA estimation errors in realistic environments and optimizes receiver deployments to be robust against various inevitable factors. Specifically, increases in DoA estimation errors caused by such factors can be amplified into localization errors depending on the receiver deployment. To mitigate this amplification, both PDoP, which quantifies the degree of error amplification depending on the geometric deployment of receivers, and DoA estimation errors are simultaneously considered. Accordingly, ray-tracing-based Wireless InSite simulations incorporating terrain and buildings are conducted to account for DoA estimation errors arising in practical environments, including multipath propagation [33]. Based on the simulation model, a cost function that simultaneously incorporates DoA estimation errors at each receiver and PDoP is formulated, and a Genetic Algorithm (GA) is applied to minimize this cost function to determine the optimal receiver deployment. To validate the localization performance, the optimized receiver deployment is compared with deployments obtained using optimization methods based on different criteria. Robustness tests are also conducted under additional DoA estimation errors in practical propagation environments. These results demonstrate that the proposed method provides reliable localization performance in realistic environments while remaining robust against increasing DoA estimation errors.

## 2. Jamming Scenario Modeling and Optimal Receiver Deployment

### 2.1. Localization Error Analysis Considering Receiver Deployment

**Figure 1:**
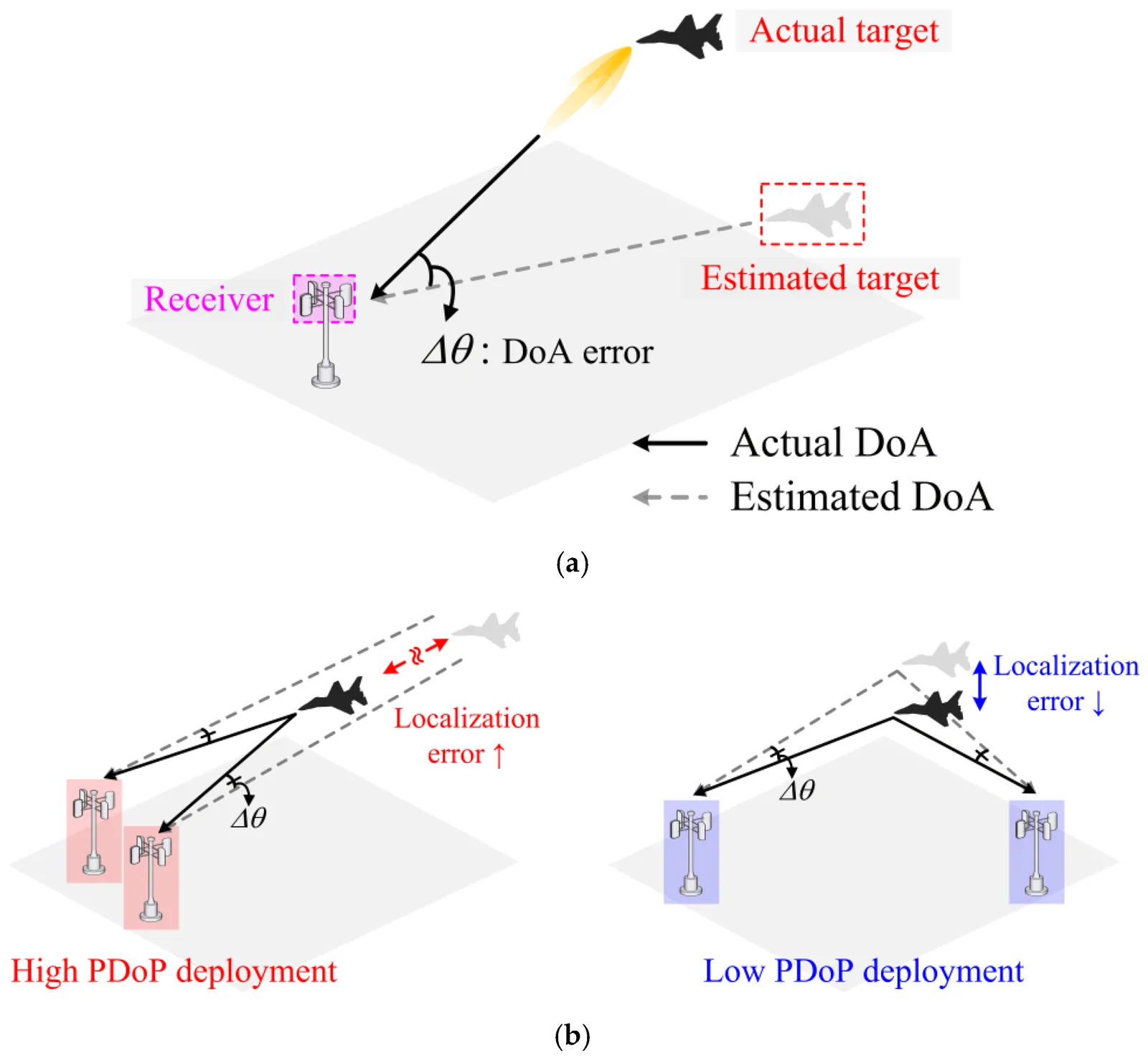
Impact of DoA estimation errors and receiver deployment on localization accuracy: (**a**) DoA estimation errors at a single receiver; (**b**) Effect of receiver deployment on localization accuracy in AoA-based systems.

Figure 1 illustrates the impact of DoA estimation errors and receiver deployment on localization accuracy. In an AoA-based localization system, multiple receivers estimate DoAs and determine the target position through triangulation, where the localization performance depends on the DoA estimation accuracy at each receiver and the receiver deployment. As shown in Figure 1a, DoA estimation errors inevitably arise at a single receiver, which can lead to localization errors between the actual and estimated target positions. These DoA estimation errors arise from two primary sources in practical environments. One is environmentally induced DoA estimation error caused by multipath propagation due to reflections and diffractions from terrain and buildings. The other is receiver-related DoA estimation error, which arises from factors such as array imperfections, mutual coupling, and processing errors. During the DoA estimation process, each receiver is affected by these factors, resulting in a combined DoA estimation error (*Δθ*). Furthermore, localization accuracy in an AoA-based system is influenced not only by the DoA estimation error but also by the geometric deployment of the receivers, as illustrated in Figure 1b. The degree of localization error varies depending on the receiver deployment, even with the same DoA estimation error, as quantified by the PDoP. The corresponding PDoP for a given target position and receiver deployment is expressed in Equations (1)–(3):

(1) $$\phi_{m}=\tan^{-1}\left(\frac{y_{\mathrm{Target}}-y_{{\mathrm{Receiver}}_{m}}}{x_{\mathrm{Target}}-x_{{\mathrm{Receiver}}_{m}}}\right)$$

(2) $$\theta_{m}=\tan^{-1}\left(\frac{z_{\mathrm{Target}}-z_{{\mathrm{Receiver}}_{m}}}{\sqrt{{\left(x_{\mathrm{Target}}\;-\;x_{{\mathrm{Receiver}}_{m}}\right)}^{2}\;+\;{\left(y_{\mathrm{Target}}-y_{{\mathrm{Receiver}}_{m}}\right)}^{2}\;}}\right)$$

(3) $$H=\left[\begin{array}{ccc} \frac{\partial \phi_{1}}{\partial x} & \frac{\partial \phi_{1}}{\partial y} & \frac{\partial \phi_{1}}{\partial z}\\ \frac{\partial \theta_{1}}{\partial x} & \frac{\partial \theta_{1}}{\partial y} & \frac{\partial \theta_{1}}{\partial z}\\ \vdots & \vdots & \vdots \\ \frac{\partial \phi_{m}}{\partial x} & \frac{\partial \phi_{m}}{\partial y} & \frac{\partial \phi_{m}}{\partial z}\\ \frac{\partial \theta_{m}}{\partial x} & \frac{\partial \theta_{m}}{\partial y} & \frac{\partial \theta_{m}}{\partial z} \end{array}\right]$$

where *ϕ_m_* and *θ_m_* denote the relative azimuth and elevation angles of the *m*-th receiver with respect to the target position, and **H** denotes the Jacobian matrix representing the sensitivity of these angles with respect to variations in the target position. From these relationships, the localization error covariance and the degree of error amplification depending on the geometric deployment can be defined as follows in Equations (4) and (5):

(4) $$Q={\left(H^{T}H\right)}^{-1}$$

(5) $$\mathrm{PDoP}=\sqrt{\mathrm{trace}\;(Q)}$$

For example, when receivers are closely deployed within a region, the estimated DoAs for a given target can lead to a high PDoP and increased localization uncertainty. Consequently, even under the same DoA estimation errors, the localization error depends on the receiver deployment, with larger PDoP values leading to significant error amplification.

### 2.2. Jamming and Receiver Modeling

**Figure 2:**
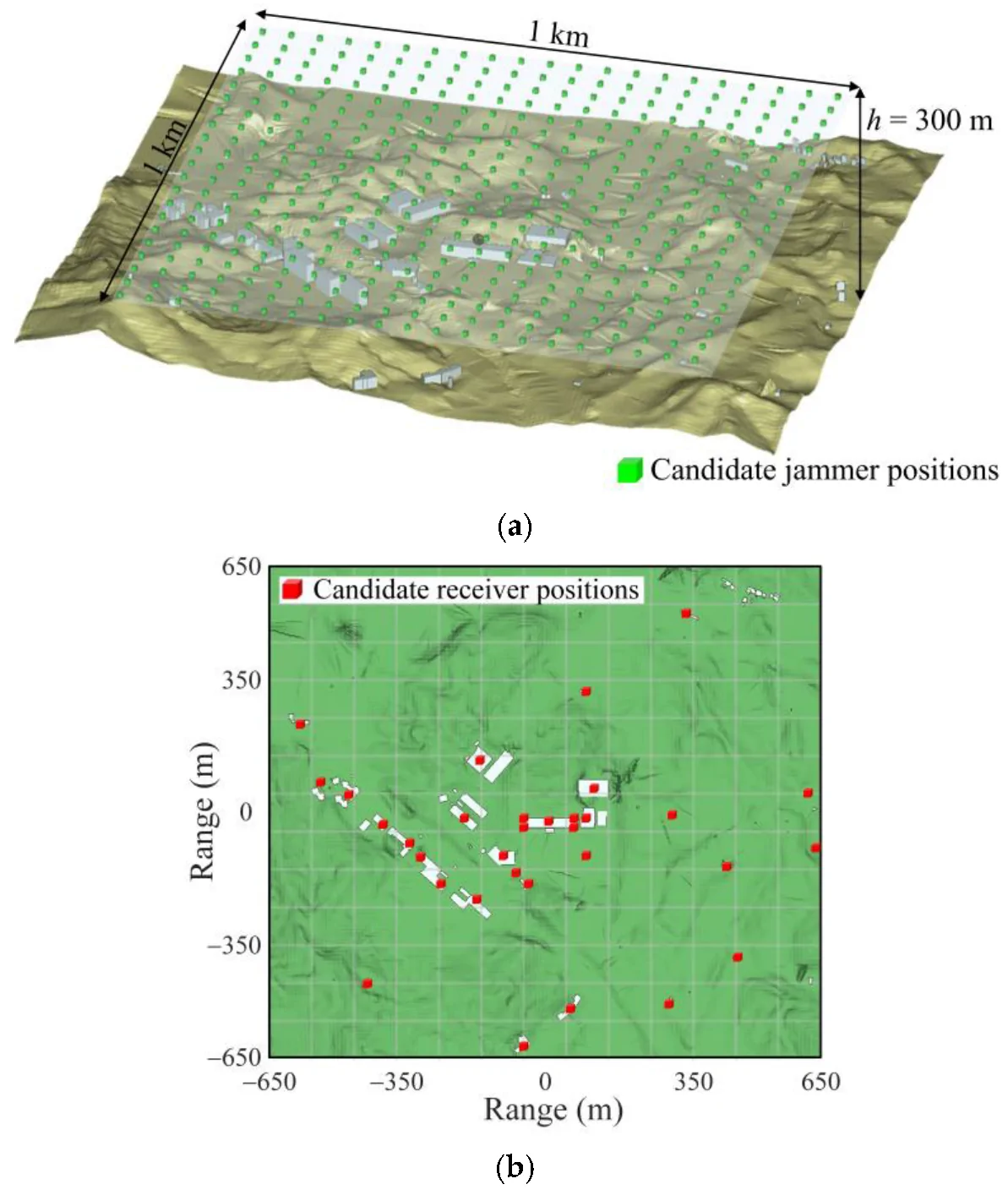
Candidate positions in the jamming scenario: (**a**) candidate jammer positions at an altitude of 300 m; (**b**) candidate receiver positions on building rooftops.

Figure 2 shows candidate jammer and receiver positions for the jamming scenario. To accurately characterize DoA estimation errors while accounting for realistic environments and receiver installation constraints, we employ the ray-tracing-based simulation tool Wireless InSite to model propagation environments including the actual terrain and buildings. The propagation environment is modeled with concrete buildings (*ε_r_* = 15, *σ* = 0.015 S/m), and wet earth ground (*ε_r_* = 25, *σ* = 0.02 S/m). As shown in Figure 2a, the candidate jammer positions (target positions) are defined over a 1 km × 1 km area at an altitude of 300 m with a grid spacing of 50 m. At each position, the jammer is assumed to emit interference signals toward the ground station. Figure 2b shows the candidate receiver positions considered for optimizing receiver deployment. A total of 32 candidate positions are placed on building rooftops within an area of approximately 1.3 km × 1.3 km surrounding the ground station. Among these, four receivers are selected to configure an AoA-based localization system.

**Figure 3:**
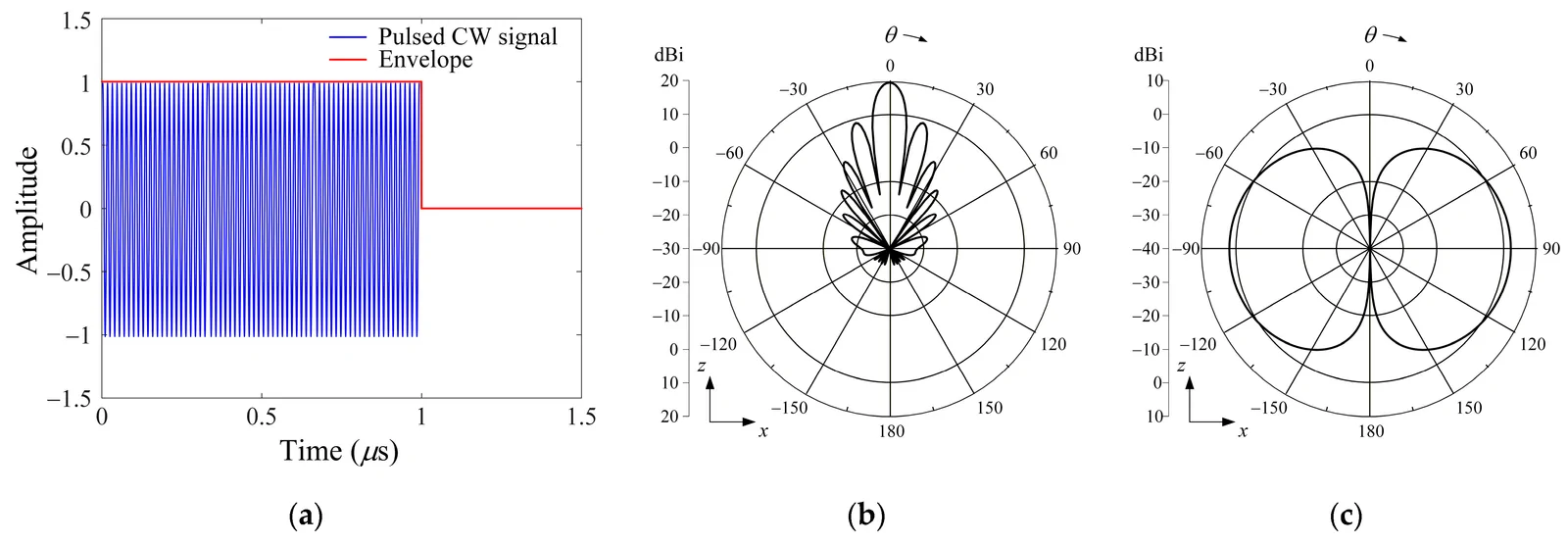
Jamming signal waveform and antenna radiation patterns in the jamming scenario: (**a**) waveform of the pulsed CW jamming signal; (**b**) radiation pattern of the horn antenna at *ϕ* = 0°; (**c**) radiation pattern of the half-wave dipole antenna at *ϕ* = 0°.

Figure 3 shows the jamming signal waveform and antenna radiation patterns in the jamming scenario. As shown in Figure 3a, the jamming waveform is modeled with a pulse width of 1 μs and a Pulse Repetition Frequency (PRF) of 1 kHz. The jamming signal is assumed to be received at each receiver with a Jamming-to-Noise Ratio (JNR) of 50 dB. In Figure 3b, the jammer is equipped with a horn antenna and emits the jamming signal toward the ground station with a transmit power of 40 dBm and an antenna gain of 18.4 dBi. Each receiver employs a Uniform Circular Array (UCA) consisting of eight half-wavelength dipole antennas with a gain of 2.15 dBi. The received signals at the UCA elements are obtained from the direct, reflected, and diffracted propagation paths calculated by Wireless InSite and are then used to construct the complex array response for DoA estimation. For each array element, the complex components of the received paths are coherently summed considering their phase information to form the complex array response vector $h={\left[h_{1},h_{2},\ldots ,h_{8}\right]}^{T}$.

A narrowband complex sinusoidal signal is then applied to h, and independent complex AWGN is added to each array channel according to the specified JNR, generating the array snapshot matrix as $X=hs^{T}+N$. The sample covariance matrix is calculated from X, followed by eigenvalue decomposition to construct the noise subspace. Finally, the DoAs at each receiver are estimated using the MUSIC algorithm with 25 snapshots by searching the azimuth and elevation directions with an angular resolution of 0.5°. The localization system estimates the jammer position through least-squares-based triangulation using the estimated DoAs. The detailed scenario parameters for the jammer and localization system are listed in Table 2.

### 2.3. Optimal Receiver Deployment Using a Genetic Algorithm

**Figure 4:**
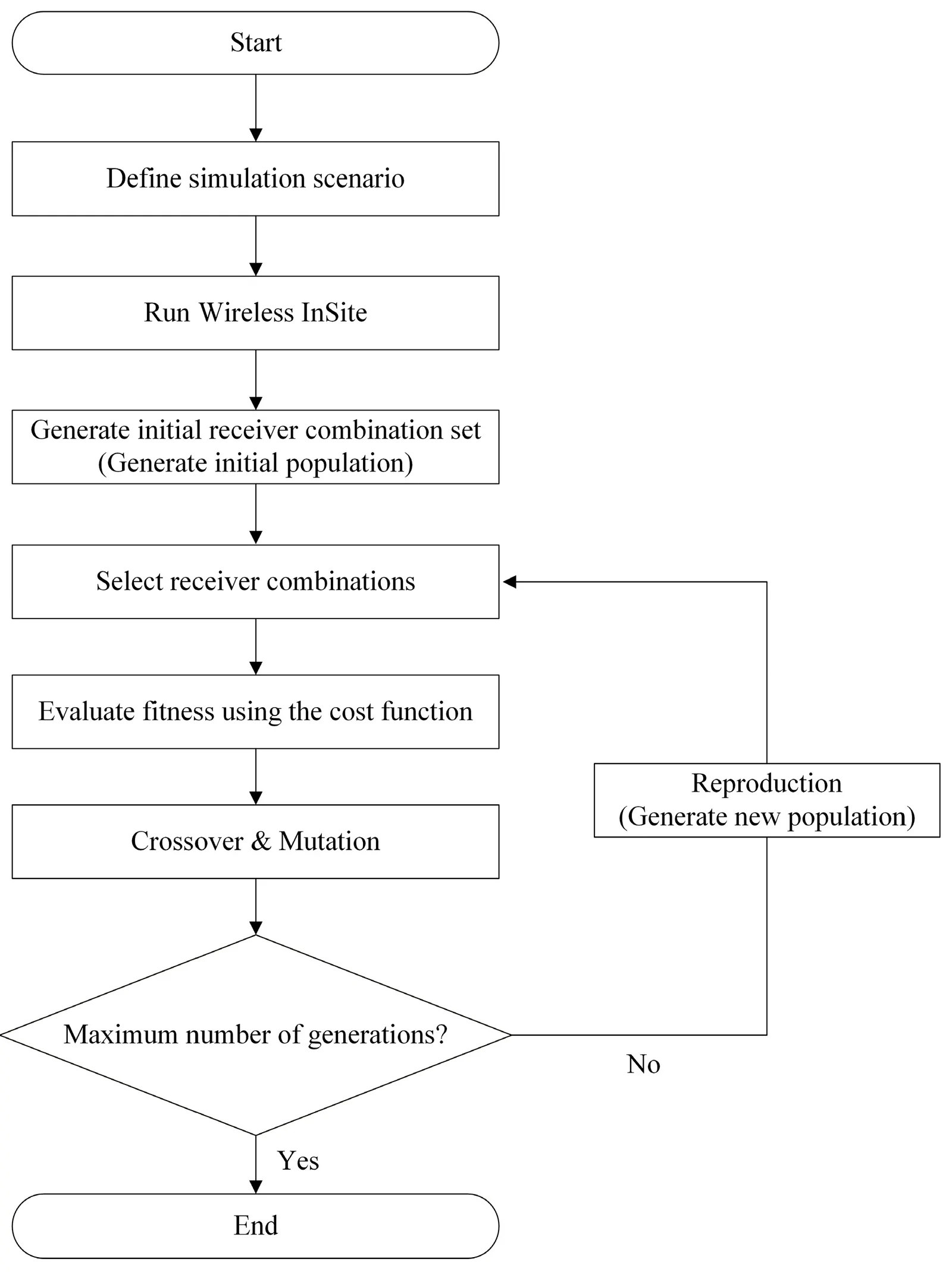
Flowchart of the GA for optimal receiver deployment.

To optimize receiver deployment for robust localization performance under DoA estimation errors, both the DoA estimation error and PDoP should be considered. In this study, a GA is employed to optimize receiver deployment by accounting for both the DoA estimation error and the PDoP, as illustrated in the optimization process in Figure 4. This GA-based optimization process begins with constructing a jamming scenario that includes the terrain, buildings, and the positions of the jammer and receivers. The received signals at each receiver under multipath propagation conditions are simulated using the ray-tracing-based simulator Wireless InSite. Based on these simulation data, the azimuth and elevation errors between the estimated and true DoAs at each receiver for a given target position are defined as follows:

(6) $$\Delta \theta_{n,m}={\hat{\theta }}_{n,m}-\theta_{n,m},\;\Delta \phi_{n,m}={\hat{\phi }}_{n,m}-\phi_{n,m}$$

where *n* is the index of the target position, *m* is the index of the receiver, and $\theta_{n,m}$ and $\phi_{n,m}$ denote the true elevation and azimuth angles from the *n*-th target position to the *m* -th receiver, respectively, while ${\hat{\theta }}_{n,m}$ and ${\hat{\phi }}_{n,m}$ are the corresponding estimated elevation and azimuth angles at that receiver.

These errors are then combined using the three-dimensional angular-error metric as follows:

(7) $$\Delta \gamma_{n,m}=\sqrt{{\left(\Delta \theta_{n,m}\right)}^{2}+{\left(\cos\theta_{n,m}\cdot \Delta \phi_{n,m}\right)}^{2}}$$

Accordingly, the DoA RMSE at the *n*-th target position is calculated as follows:

(8) $${\mathrm{DoA}\;\mathrm{RMSE}}_{n}=\sqrt{\frac{1}{M}\sum_{m=1}^{M}{\left(\Delta \gamma_{n,m}\right)}^{2}}$$

where *M* is the number of receivers constituting a system.

As expressed in Equation (5), the localization error depends on the DoA estimation error and the PDoP, where the PDoP characterizes the geometric amplification of the DoA estimation error, with larger values leading to increased localization errors. Therefore, a cost function that considers both the DoA estimation errors and the PDoP is defined in Equation (9):

(9) $$\mathrm{Cost}\;=\;\sqrt{\frac{1}{N}\sum_{n\;=\;1}^{N}{\left[{\mathrm{DoA}\;\mathrm{RMSE}}_{n}\cdot {\left({\mathrm{PDoP}}_{n}\right)}^{3}\right]}^{2}}$$

where *N* is the number of target positions, DoA RMSE*_n_* and PDoP*_n_* represent the DoA RMSE and the PDoP value for the *n*-th target position, respectively. The proposed cost function considers DoA estimation errors that may arise in realistic environments through the DoA RMSE, while simultaneously accounting for the geometric error amplification due to receiver deployment through the PDoP. In particular, by applying a higher-order weighting to the PDoP term, the cost function is designed to prevent the selection of receiver deployments with high PDoP that can significantly amplify DoA estimation errors into localization errors. This cost function mitigates excessive error amplification and enables robust localization performance even under increased DoA estimation errors. Based on the defined cost function, the GA proceeds as follows: First, an initial population is constructed by selecting subsets from the candidate receiver positions. Second, in each generation, the fitness of each receiver deployment is evaluated by computing the predefined cost function, and superior individuals are selected using a tournament selection method. Third, new populations are subsequently generated through crossover and mutation operations, and this process is iteratively repeated. In this study, the maximum number of generations and population size are set to 100 and 150, respectively. The effective mutation probability for changing the receiver combination is approximately 0.267, and the crossover probability is set to 0.3 considering the short chromosome consisting of only four receiver indices. Finally, the receiver deployment with the lowest cost value among all generations is selected as the optimal deployment.

## 3. Performance Analysis of Optimal Receiver Deployment

**Figure 5:**
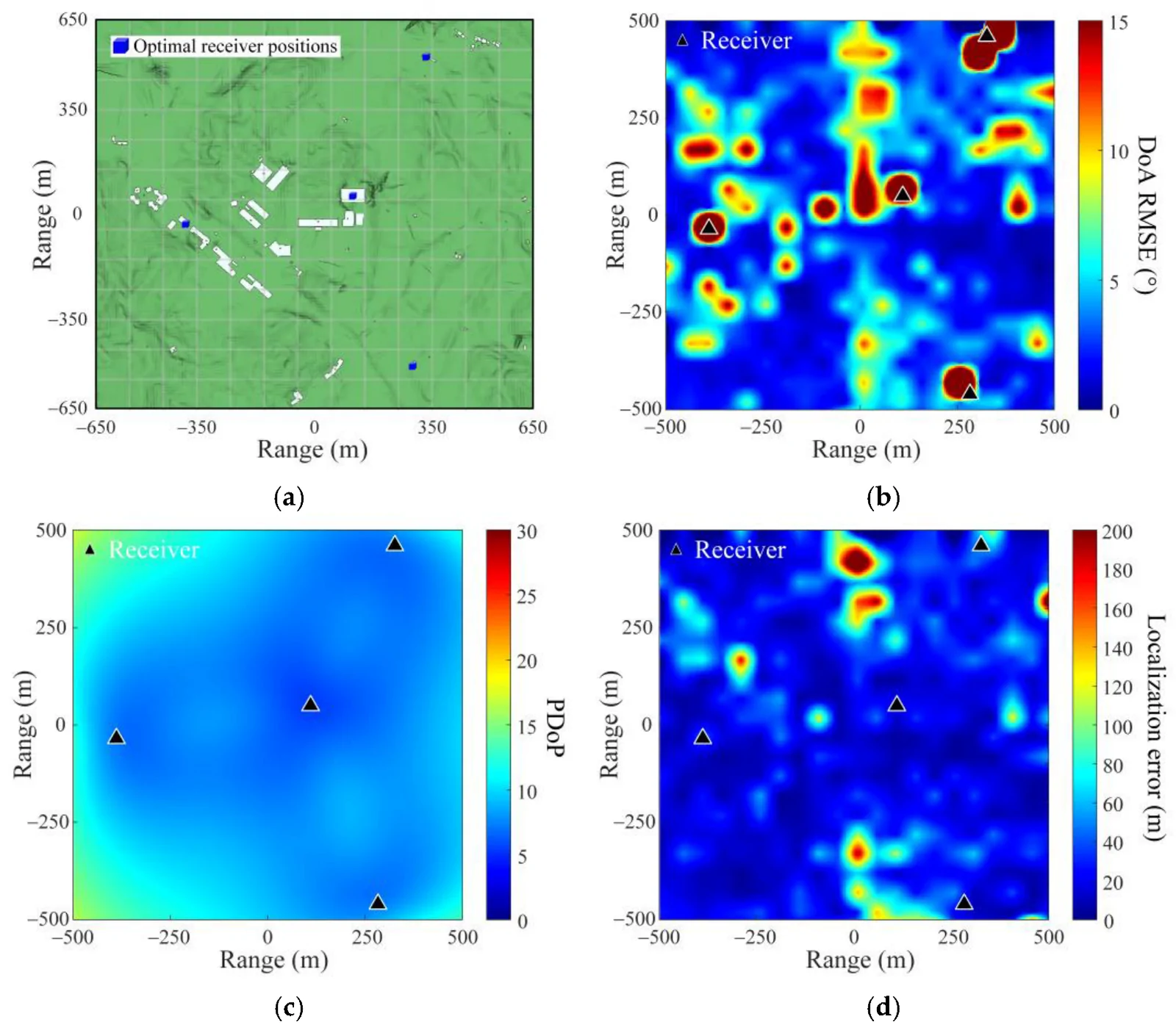
Optimal receiver deployment and corresponding performance metrics: (**a**) positions of the optimal receivers on the map; (**b**) DoA RMSE for each target position under the optimal receiver deployment; (**c**) PDoP map for target positions under the optimal receiver deployment; (**d**) localization error map for each target position under the optimal receiver deployment.

Figure 5 presents the optimal receiver deployment and the corresponding performance metrics. As shown in Figure 5a, the optimal deployment is spatially distributed on multiple buildings with different heights. This simulation environment considers not only environmental factors, such as multipath propagation, but also receiver-related DoA estimation errors, including a maximum error of approximately 1° considering the angular resolution of the DoA estimation algorithm (3D MUSIC). The DoA RMSE remains below 5° for most target positions, although it increases in certain regions, resulting in an average DoA RMSE of 8.16°, as observed in Figure 5b. This degradation can be attributed to the reduced DoA estimation accuracy for targets located near the zenith direction due to the radiation pattern characteristics of the half-wavelength dipole elements employed in the receiver array [34,35], as well as the effects of multipath propagation due to reflections and diffractions. Figure 5c illustrates the PDoP distribution for each target position. Since the receivers are deployed to surround the target positions, the PDoP remains uniformly low across most target positions, with an average of 9.20. This indicates that the proposed deployment effectively minimizes geometric error amplification. Figure 5d presents the localization error for each target position under the optimal deployment. By comparing Figure 5b,c, it can be observed that the low PDoP effectively mitigates the impact of relatively large DoA RMSE values on the localization error. For instance, although the DoA RMSE is relatively high for targets near the zenith direction, the corresponding localization error remains low. This is because the receiver deployment prevents significant amplification of individual DoA estimation errors into localization errors. As a result, the localization errors remain low across most target positions, with an overall localization RMSE of 51.07 m.

For quantitative benchmarking, all 35,960 possible receiver deployments are evaluated. Their mean localization RMSE and PDoP are 167.70 m and 16.27, respectively, whereas the proposed deployment achieves 51.07 m and 9.20, outperforming 98.18% and 99.93% of all deployments, respectively. In contrast, its DoA RMSE of 8.16° is slightly higher than the overall mean of 7.83°. These results show that the proposed deployment achieves a low localization RMSE despite a slightly higher DoA RMSE, highlighting the importance of considering both DoA estimation error and PDoP in receiver deployment.

**Figure 6:**
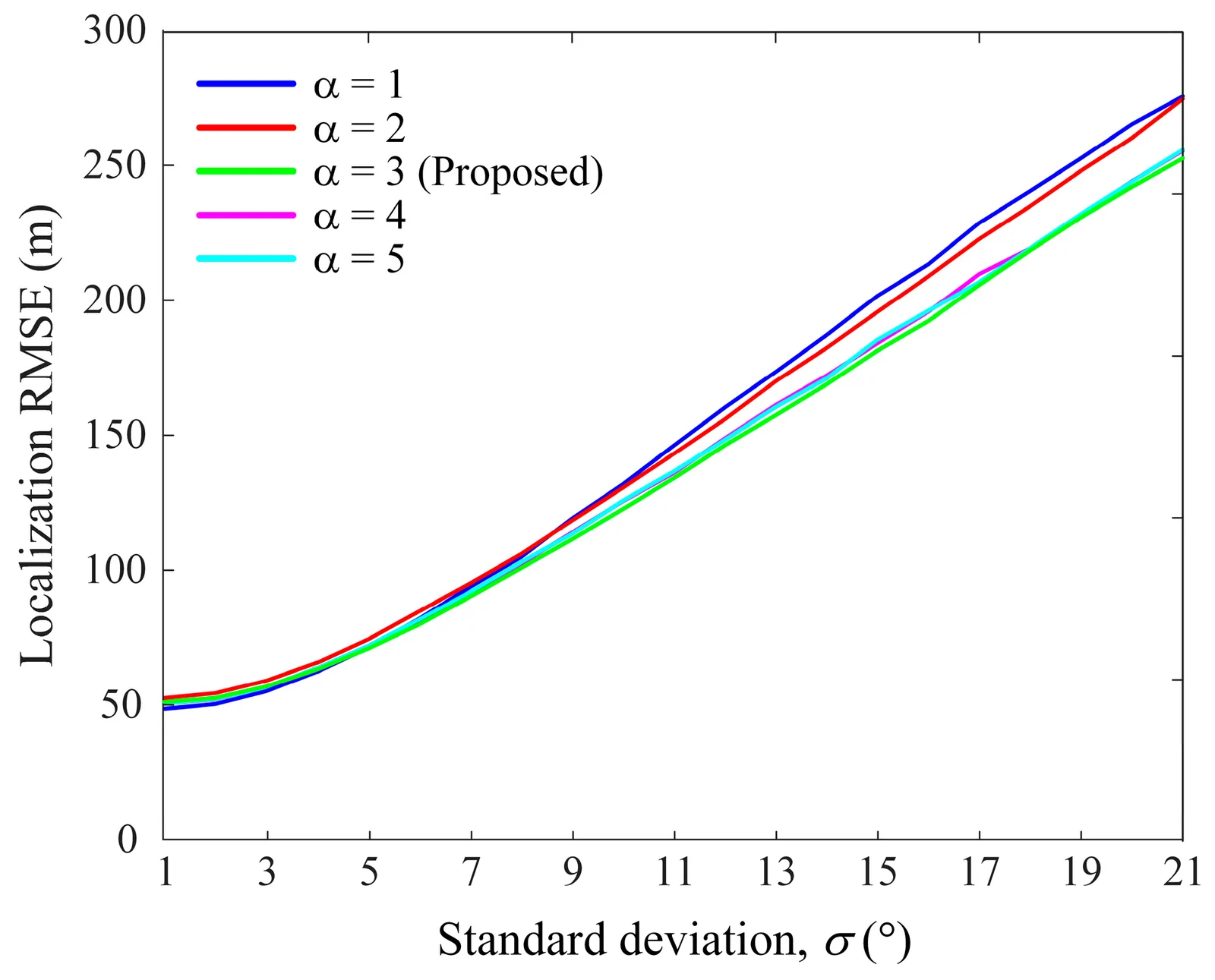
Sensitivity of localization RMSE to the PDoP weighting coefficient α under increasing receiver-related DoA estimation error.

Figure 6 shows the sensitivity of the localization RMSE to the PDoP weighting coefficient α, where the standard deviation (*σ*) of the receiver-related DoA estimation error is varied from 1° to 21°. When the additional DoA estimation error is small, the localization performance is comparable for all α values. As the error increases, however, α = 3 maintains lower localization RMSE than α = 1 and 2, indicating improved robustness against localization error amplification. In addition, α = 3 achieves a lower mean localization RMSE over the increased-error conditions than those obtained with α = 4 and 5. Based on these results, α = 3 was selected as the PDoP weighting coefficient because it provides robust localization performance under increasing DoA estimation errors.

**Figure 7:**
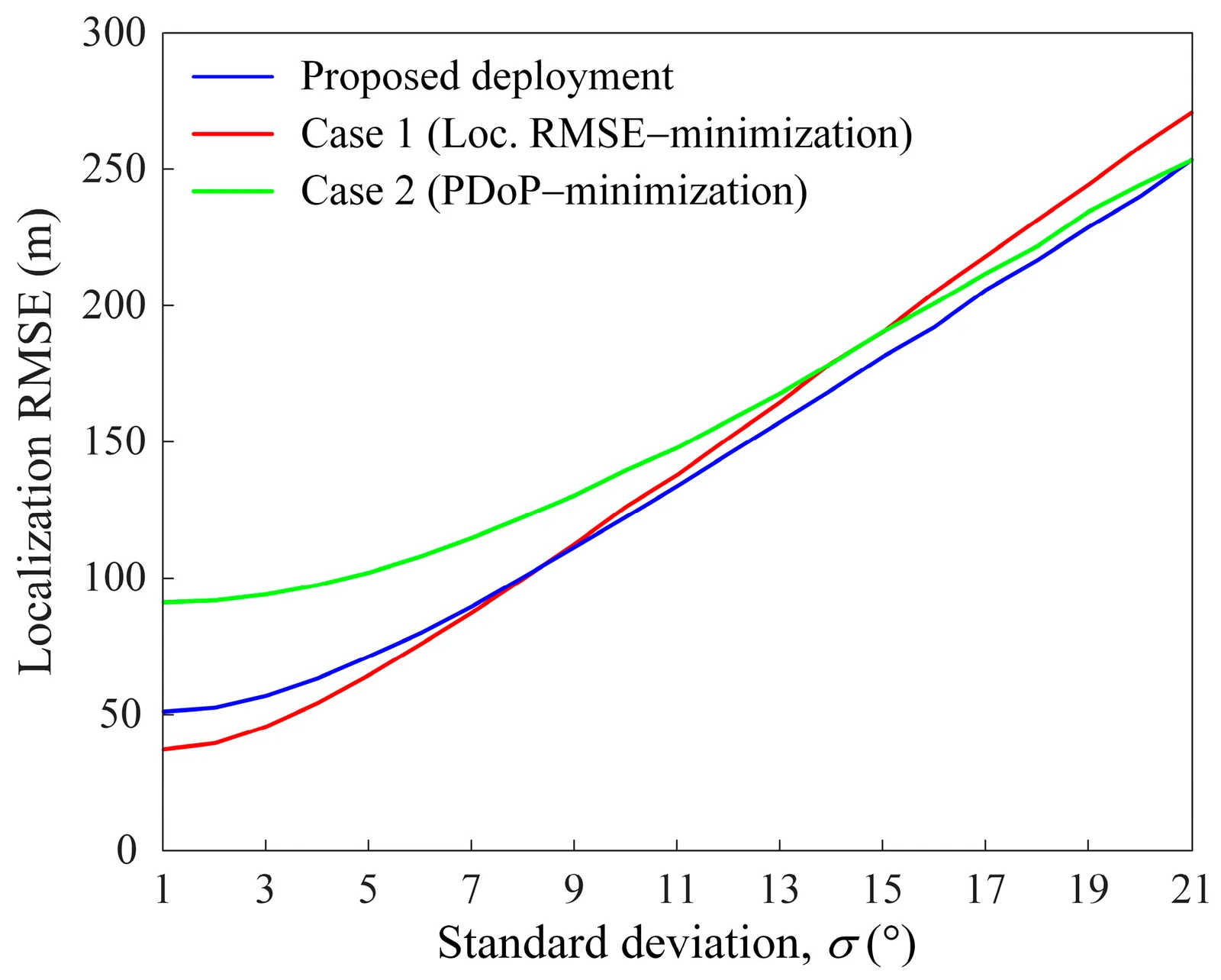
Localization RMSE for Case 1, Case 2, and proposed deployments by varying the receiver-related DoA estimation error.

Figure 7 illustrates the localization RMSE for receiver deployments optimized based on different criteria, where the standard deviation (*σ*) of the receiver-related DoA estimation error is varied from 1° to 21°. To evaluate the robustness of the proposed deployment, two additional optimized deployments are considered for comparison. Case 1 is obtained based solely on the minimization of localization RMSE (Loc. RMSE), whereas Case 2 is obtained based solely on the minimization of PDoP. The results indicate that, when the receiver-related DoA estimation error is small (approximately 1°), Case 1 achieves the lowest localization RMSE, while the proposed deployment exhibits comparable performance. In contrast, Case 2 exhibits a higher localization RMSE, because it does not account for the DoA estimation errors arising in practical propagation environments. However, as the standard deviation of the receiver-related DoA estimation error increases from approximately 8° up to 21°, Case 1 shows a rapid increase in localization error due to its higher PDoP. In contrast, both Case 2 and the proposed deployment exhibit a relatively small degree of error amplification due to their low PDoP. In particular, the proposed deployment achieves lower localization RMSE than Case 1 when the standard deviation of the receiver-related DoA estimation error exceeds approximately 8°. Overall, these results demonstrate that the proposed deployment method provides reliable localization performance in realistic propagation environments and remains robust even under increasing DoA estimation errors.

## 4. Conclusions

In this paper, we proposed a receiver deployment optimization method that ensures robust localization performance under DoA estimation errors arising in practical environments. To ensure robustness, the proposed method accounted for DoA estimation errors in realistic environments and optimized receiver deployments by incorporating PDoP to mitigate their amplification into localization errors. To accurately characterize DoA estimation errors under realistic propagation environments and installation constraints, the ray-tracing-based simulator Wireless InSite was employed to model a realistic environment including actual terrain and buildings. Based on this model, a cost function that incorporated both the DoA estimation errors at each receiver and PDoP was defined, and a GA was applied to minimize this cost function and determine the optimal receiver deployment. In realistic environments, the optimized receiver deployment prevented significant amplification of individual DoA estimation errors into localization errors, resulting in low localization errors across most target positions, with an overall localization RMSE of 51.07 m. To evaluate the robustness of the proposed deployment, two additional optimized deployments were considered for comparison. The results indicated that, when the receiver-related DoA estimation error was small, the proposed deployment exhibited comparable performance to the localization RMSE-minimizing deployment. In contrast, the PDoP-minimizing deployment exhibited a higher localization RMSE, because it did not account for the DoA estimation errors arising in practical propagation environments. In particular, the proposed deployment achieved lower localization RMSE than the localization RMSE-minimizing deployment when the standard deviation of the receiver-related DoA estimation error exceeded approximately 8°. These results demonstrated that the proposed deployment method provides reliable localization performance in realistic propagation environments and remains robust against increasing DoA estimation errors.

## Figures and Tables

**Table 1:** Comparison of representative receiver deployment approaches.

Properties	Conventional GDOP/PDoP Placement [30]	Localization Error Minimization [31]	Prior Environment-Aware Placement Methods [32]	Proposed Method
Localization method	ToA/AoA	Beacon-based	ToA-based	3-D AoA triangulation
Environment-induced measurement error	No	No	Yes (Ranging error)	Yes (DoA error)
Geometry weighting	DOP	No	DOP	PDoP^α^
Incorporation of practical DoA error and receiver geometry in optimization	No	No	No	Yes
Localization performance and robustness under increasing DoA errors	No	No	No	Yes

**Table 2:** Scenario parameters for the jammer and localization system.

	Parameters	Values
Jammer location	Grid spacing (m)	50
	Altitude (m)	300
Jamming system	Jammer antenna type	Horn
	Jamming power (dBm)	40
	Jamming frequency band	S-band
	Signal type	Pulsed CW
	Pulse width (μs)	1
	Pulse repetition frequency (kHz)	1
	JNR (dB)	50
Localization system	Number of receivers	4
	Receiver antenna type	Half-wave dipole
	Receiver array type	UCA
	Number of array elements	8
	Receiver sensitivity (dBm)	−130
Localization algorithm	Localization method	LS triangulation
	DoA estimation method	MUSIC
	MUSIC angular resolution (°)	0.5
	Snapshots	25

## Data Availability

Data are contained within the article.
